# Trueness and precision of facial scan and virtual patient representation workflow

**DOI:** 10.1111/jopr.14024

**Published:** 2025-01-21

**Authors:** Khaled Q. Al Hamad, Jad Q. Ayyad, Bashar A. Al‐Rashdan, Firas A. Al Quran

**Affiliations:** ^1^ College of Dental Medicine, QU Health Qatar University Doha Qatar; ^2^ Department of Prosthodontics Jordan University of Science & Technology Irbid Jordan; ^3^ Private practice Amman Jordan

**Keywords:** accuracy, digital dentistry, digital patient, facial scanning, scanners, structured light

## Abstract

**Purpose:**

To investigate the feasibility and accuracy (trueness and precision) of facial scanning and virtual patient representation (VPR).

**Materials and Methods:**

One participant was recruited and informed consent was obtained. VPR was performed 30 times with a custom fabricated intraoral scan body (ISB). Thirteen adhesive markers were added to the face as an extraoral scan body (ESB). Two facial scans were obtained for each VPR using an infra‐red laser accessory sensor (Structure sensor; Occipital Inc) mounted on a computer tablet (iPad Pro; Apple Inc), including one with seated ISB and one without ISB. Two maxillary intraoral scans were obtained using an intraoral scanner (Omnicam; Dentsply Sirona) with and without the ISB. All files were imported to a dental software program (exocad; exocad GmbH) and VPR was obtained by aligning the facial and IOS scans using the ISB and ESB as common elements for alignment. Five fiducial face landmarks, four intraoral dental landmarks, and six perioral landmarks were selected for measurements. A total of 32 linear measurements, including 14 face‐face (for facial scan accuracy) and 18 face‐dental (for VPR accuracy) representing total face, lower face, and perioral regions, were performed directly using a digital caliper (FINO Digital Caliper; FINO GmbH) and virtually on the VPR. Trueness was evaluated by mean absolute difference (MAD) between the virtual and direct measurements, and the standard deviation represented Precision. Statistical analyses were performed with a statistical software package (IBM SPSS Statistics v25; IBM Corp), with α = 0.05. Data were analyzed for normality with Shapiro‐Wilk test, and 1‐sample t‐ (or Wilcoxon signed rank test), technical error of measurement (TEM), and relative error magnitude (REM).

**Results:**

The facial scan had 2.04, 1.66, 0.8 trueness, and 1.05, 0.92, 0.91 precision for total, lower face, and perioral regions. VPR had higher MAD (lower trueness) than facial scan, including 3.32, 2.40, 1.21 trueness and 2.2, 1.47, 1.2 precision for total, lower face, and perioral regions. Both TEM and REM were lowest for the perioral region and increased with increasing measurement distance.

**Conclusion:**

Error in face scanning increased with increased distance and intricate details. VPR accuracy was lower than face scan accuracy because of added errors in the alignment process. The investigated VPR workflow might be feasible for treatment planning and smile design. However, it would be unreliable for more demanding prostheses manufacturing purposes.

Obtaining digital representations of patients is gaining popularity in the dental literature.^1^, ^2^ The virtual patient representation (VPR) constitutes the integration of the patient's digital information, including cone beam computed tomography (CBCT), intraoral scans (IOS), and facial scans (FS).^3^, ^4^ The CBCT and IOS digital files are aligned by using fiducial markers as common elements, whereas IOS and FS files are aligned with extraoral and intraoral scan bodies.^1^, ^2^, ^3^, ^4^ The intraoral scan body (ISB) acts as a common element between the IOS and the FS, whereas the extraoral scan body (ESB) acts as a common element between the FS with ISB and the reference FS without ISB.^1^, ^2^, ^3^, ^4^ ISBs could be obtained from different manufacturers, or could be independently designed and additively manufactured.^3^, ^4^, ^5^ Also, ESB could be replaced with marks placed on the forehead and nose bridge.^5^

Noncontact facial scanners with image‐based or range‐based techniques are commonly used to obtain FSs.^6^ Image‐based scanners with stereophotogrammetry technology combine two or more images made from different angles and have been considered the reference standard.^7^ These stationary scanners offer instantaneous capture time, life‐like rendering, and high accuracy.^7^ However, these scanners demand extensive set‐up and calibration, space consumption, complex workflow, and high cost.^6^, ^7^ In contrast, range‐based facial scanners use structured light technology and offer several advantages, including comparable accuracy, mobility, and no space consumption. Nevertheless, they are still relatively expensive and are commonly handheld requiring considerable training.^8^ Accessory sensors using structured light technology have been introduced for mobile smart phones and tablet computers for FS purposes. These accessories offer potentially cheaper alternatives and user‐friendly workflows.^3^, ^8^, ^9^

Several studies investigated the accuracy of VPR by using different digital data, face scanners, and workflows.^6^, ^8^, ^9^, ^10^, ^11^, ^12^, ^13^, ^14^, ^15^, ^16^, ^17^ Nevertheless, there is no consensus on the best protocol for obtaining a VPR. Also, studies investigating the accuracy of facial scanning with accessory sensors mounted on tablet computers are scarce. Therefore, the purpose of this study was to investigate the accuracy and feasibility of facial scanning with an accessory scanner sensor, and the accuracy and feasibility of VPR obtained with intraoral and facial scan alignment. The first null hypothesis was that there would be no observed difference between the virtual and direct face dimension measurements (FS accuracy). The second null hypothesis was that there would be no observed difference between the VPR and direct face‐dental dimension measurements (VPR workflow accuracy).

## MATERIALS AND METHODS

**FIGURE 1:**
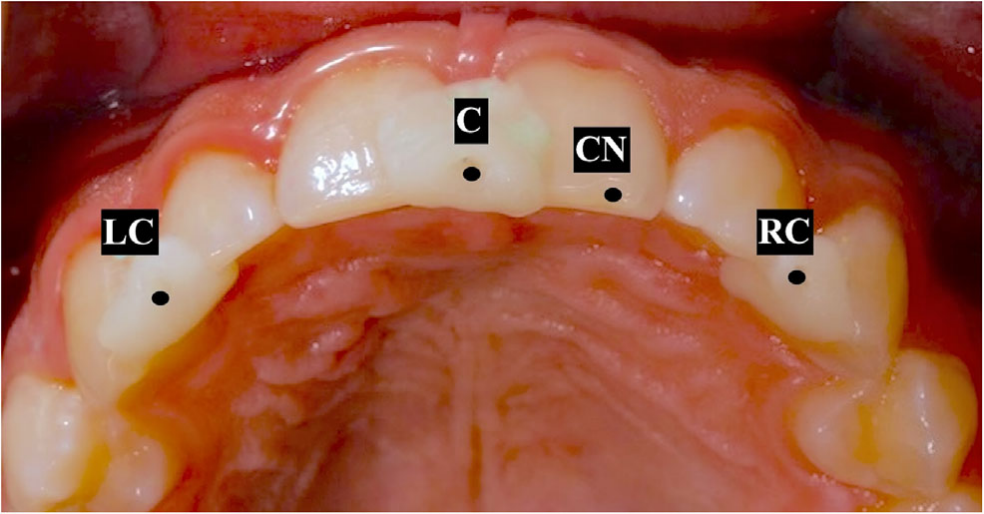
Indentation creation with composite resin addition in maxillary embrasure areas for instrument stabilization during the direct measurement. (C) Incisal embrasure between central incisors, (LC) incisal embrasure between left lateral incisor and left canine, (RC) incisal embrasure between right lateral incisor and right canine, (CN) notch on central incisor.

**FIGURE 2:**
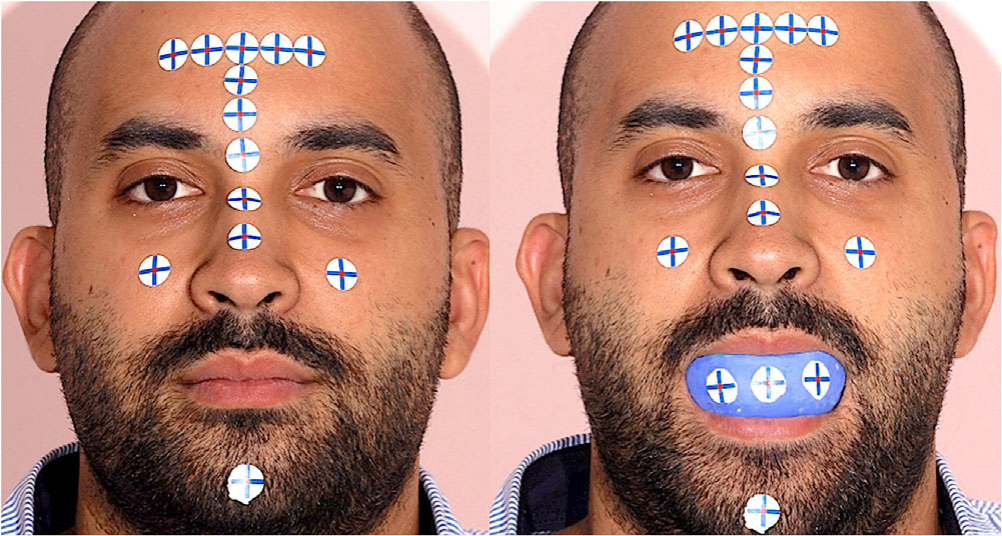
Extraoral scan body (ESB) with 13 custom‐designed adhesive markers (image left), and intraoral scan body (ISB) with silicone guide and three custom‐designed adhesive markers (image right).

**FIGURE 3:**
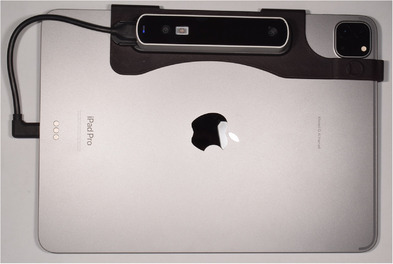
Mounted accessory scanner (Structure sensor pro; Occipital Inc) on tablet computer (iPad Pro; Apple Inc).

**FIGURE 4:**
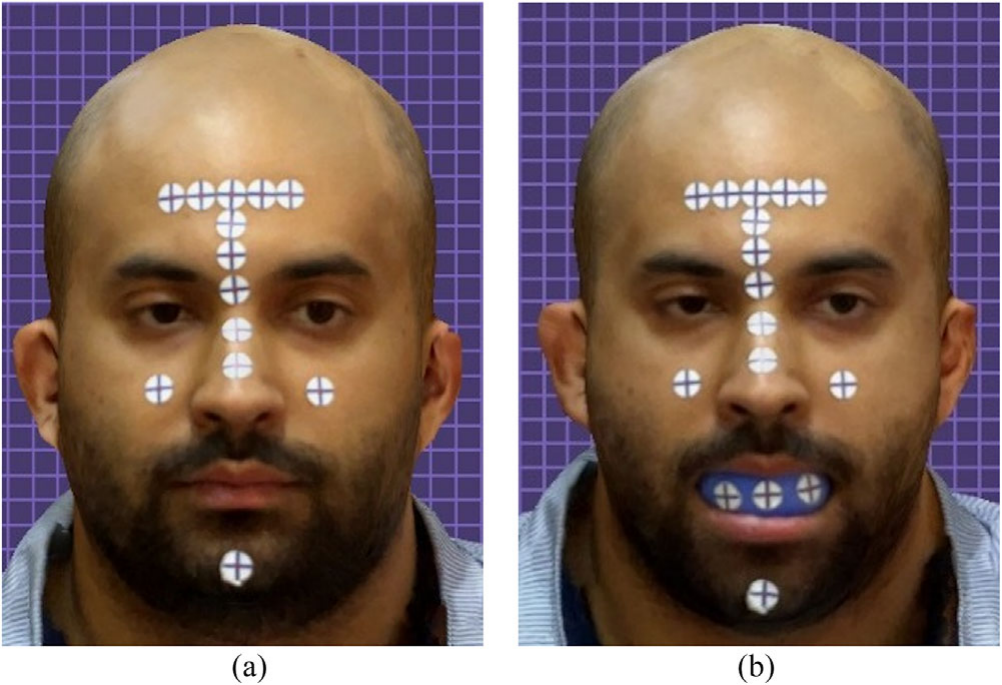
Facial scans. (a) Facial scans without silicone guide intraoral scan body. (b) Facial scans with silicone guide intraoral scan body.

**FIGURE 5:**
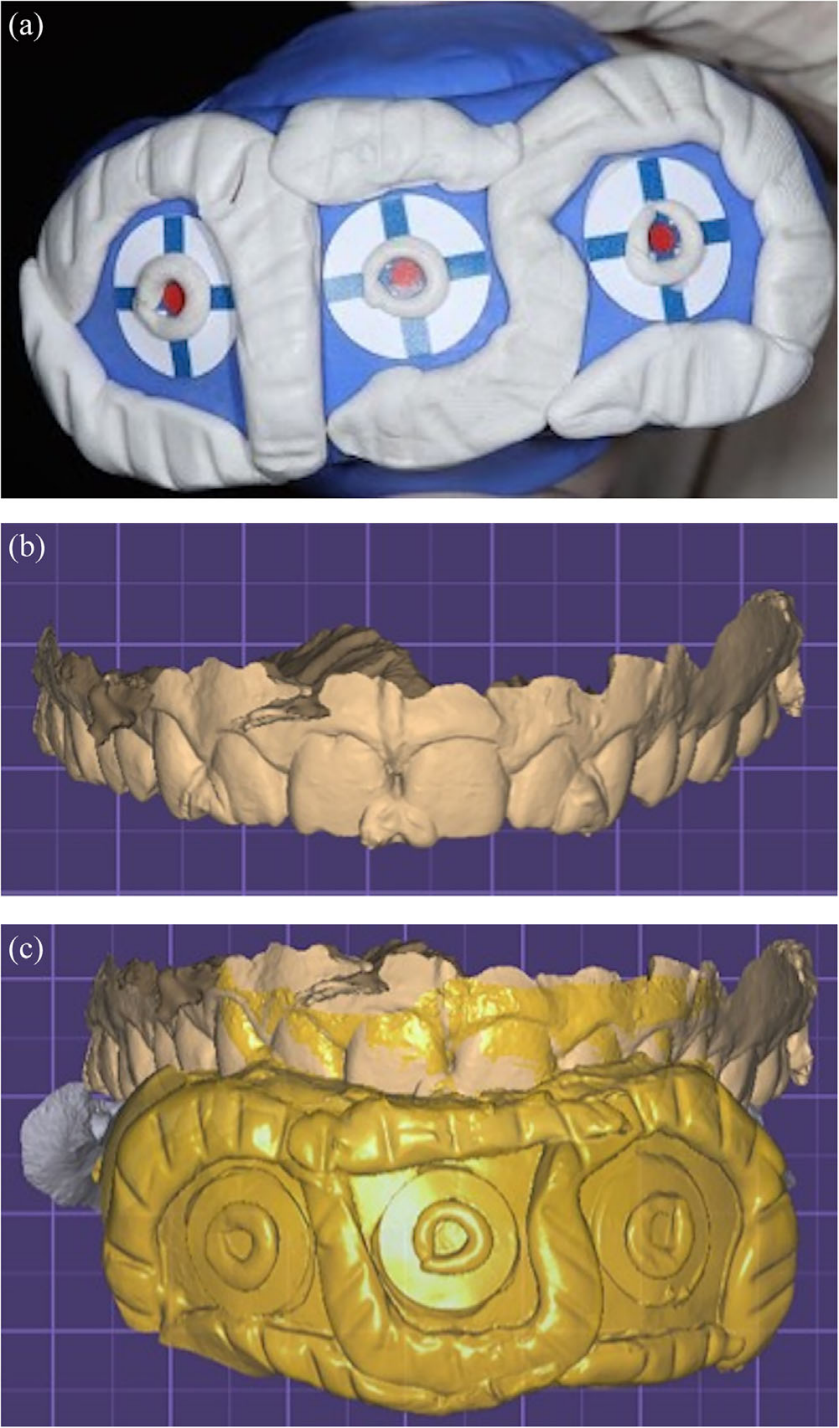
Intraoral scans (IOS) with and without the intraoral scan body (ISB). (a) Formation of distinct features on ISB with adhesive putty. (b) Maxillary IOS without ISB. (c) IOS with ISB and IOS without ISB aligned.

**FIGURE 6:**
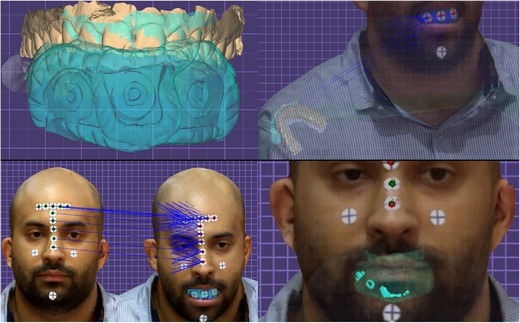
Alignment workflow for virtual patient representation (VPR). (Step 1: Top left): Alignment of intraoral scans (IOSs) with and without silicone guide intraoral scan body (ISB). (Step 2: Top right): Alignment of files in step 1 to facial scan with ISB using ISB as common element. (Step 3: Bottom left): Alignment of files in step 2 to facial scan without ISB using 13 markers as common elements. (Step 4: Bottom right): Achievement of VPR with all files aligned. Files in between IOS without ISB and facial scan without ISB can now be unchecked to hide them.

Approval for this in vivo study was obtained from the Institutional Review Board (IRB 69‐140‐2021) and the local scientific committees (ID20230308) of Jordan University. A sample size of 28 was determined with a study power: 0.9, α: 0.10, and effect size> 0.50 (G*Power Ver. 3.0.10; Universität Kiel, Germany). One 27‐year‐old male subject was recruited and informed consent was obtained. The subject had complete maxillary dentition and no maxillofacial tumors or deformities. Three maxillary intraoral landmarks were created with composite resin (3M Filtek; 3M, Irvine, California, USA) in the incisal embrasure between central incisors (C), incisal embrasure between left lateral incisor and left canine (LC), and incisal embrasure between right lateral incisor and right canine (RC) (Figure 1). The resin composite was used to shape the embrasure areas with the end of a caliper tip instrument and create an indentation for stabilization during the direct measurement. The VPR was performed 30 times for the same subject with a workflow that was adopted from a previous study.^3^ A custom silicon guide was fabricated to act as the ISB using putty polyvinyl siloxane impression material [elite HD+ putty soft; Zhermack, Badia Polesine (RO), Italy]. The subject was asked to occlude gently over the mixed putty and embed the incisal third of the maxillary anterior teeth. The dimensions of the silicone guide were made to accommodate three custom‐designed sticky markers with 12 mm diameter that were placed on the labial surface of the silicone guide ISB. Another 13 markers were also placed on the face to act as the ESB: 10 on non‐mobile tissues in the forehead and bridge of the nose, 2 markers on the cheeks, and 1 on the chin (Figure 2).^3^, ^4^, ^5^ Facial scans were obtained by using a range‐based sensor (Structure sensor pro; Occipital Inc, Colorado, USA) mounted on a computer tablet (iPad Pro; Apple Inc, California, USA) (Figure 3). The sensor uses infra‐red laser beam with Class 1 safety.^3^, ^11^ The facial scanning was performed in a room with diffuse 65 k ambient lighting and with the curtains closed. The subject was seated on an adjustable chair, maintaining a natural head position. Face scanning was performed by following a circular path around the head with a distance guided by a dedicated scanning software program (ItSeez3D App; ItSeez3D, California, USA). The software provided visual scanning instructions with color and written guidance. One FS was made with the ISB and another without the ISB and with the patient at maximum intercuspal position. This process was repeated 30 times and a total of 30 FSs with the ISB and 30 without the ISB were made. All FSs were constructed using the scanning software program (ItSeez3D App; ItSeez3D) and were exported in Object File Format (OBJ) (Figure 4a, 4b). The ISB silicone guide was modified by placing adhesive putty (Faber‐Castell; BhD, Stein, Germany) and shaped by forming distinct features. Two IOSs of the maxillary arch were made: one with and one without the ISB silicone guide (Omnicam; CEREC, Dentsply Sirona, York, Pennsylvania, USA). The two files were aligned using the IOS software and then exported in STL file format. The IOS files were imported to a dental CAD software program (exocad; exocad GmbH, Darmstadt, Germany) (Figure 5a‐c). The aligned IOS files were then aligned to the 30 FSs with and 30 FSs without the ISB to provide 30 VPRs according to the protocol used in previous studies (Figure 6).^3^, ^4^, ^5^

**FIGURE 7:**
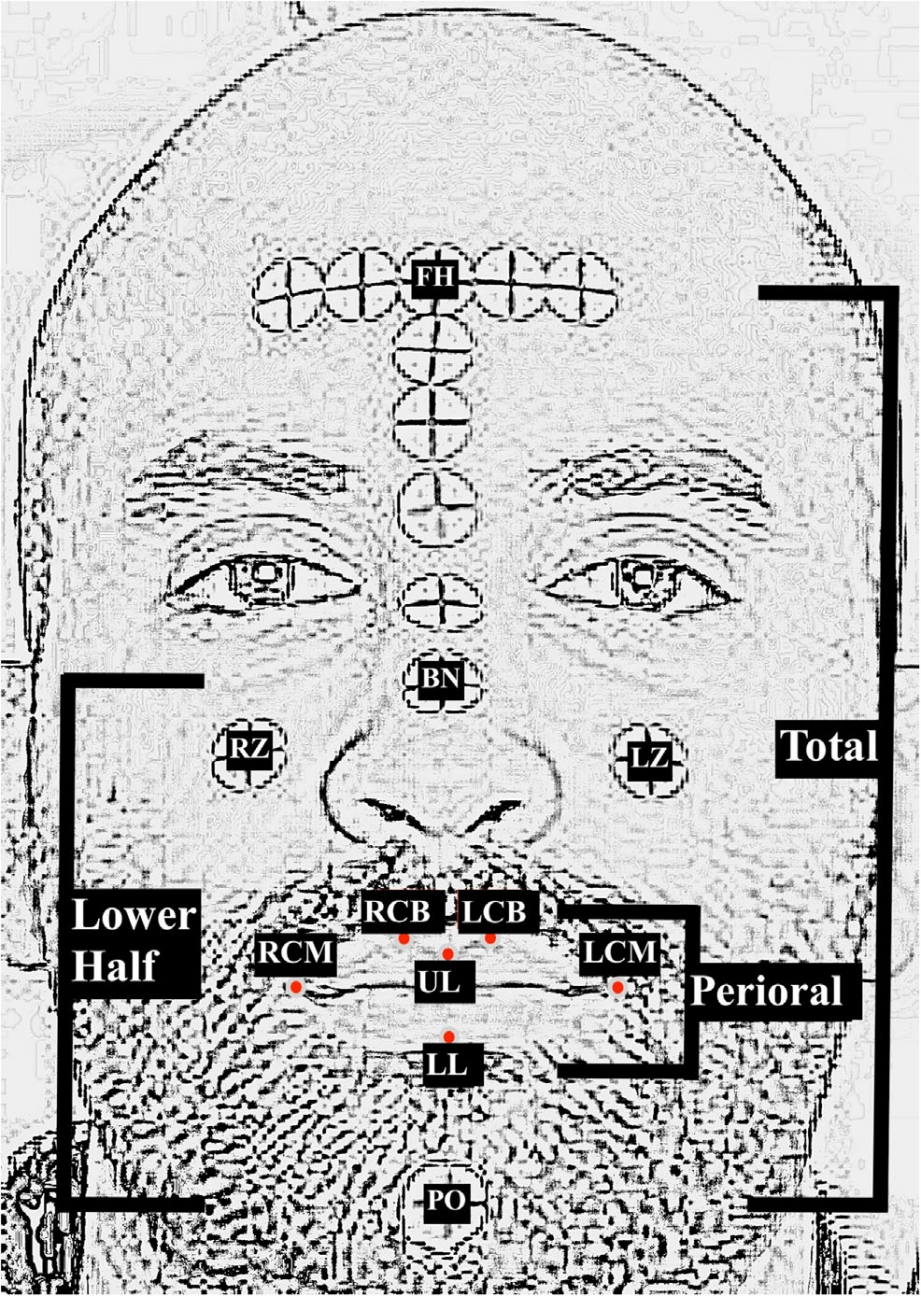
Selected facial landmarks and regions. BN, bridge of nose; FH, middle of forehead; LCM, left commissure; LCB, left cupid bow; LL, middle of vermilion of mandibular lip; LZ, left zygon; PO, pogonion; RCB, right cupid bow; RCM, right commissure; RZ, right zygon; UL, middle of vermilion of maxillary lip.

**FIGURE 8:**
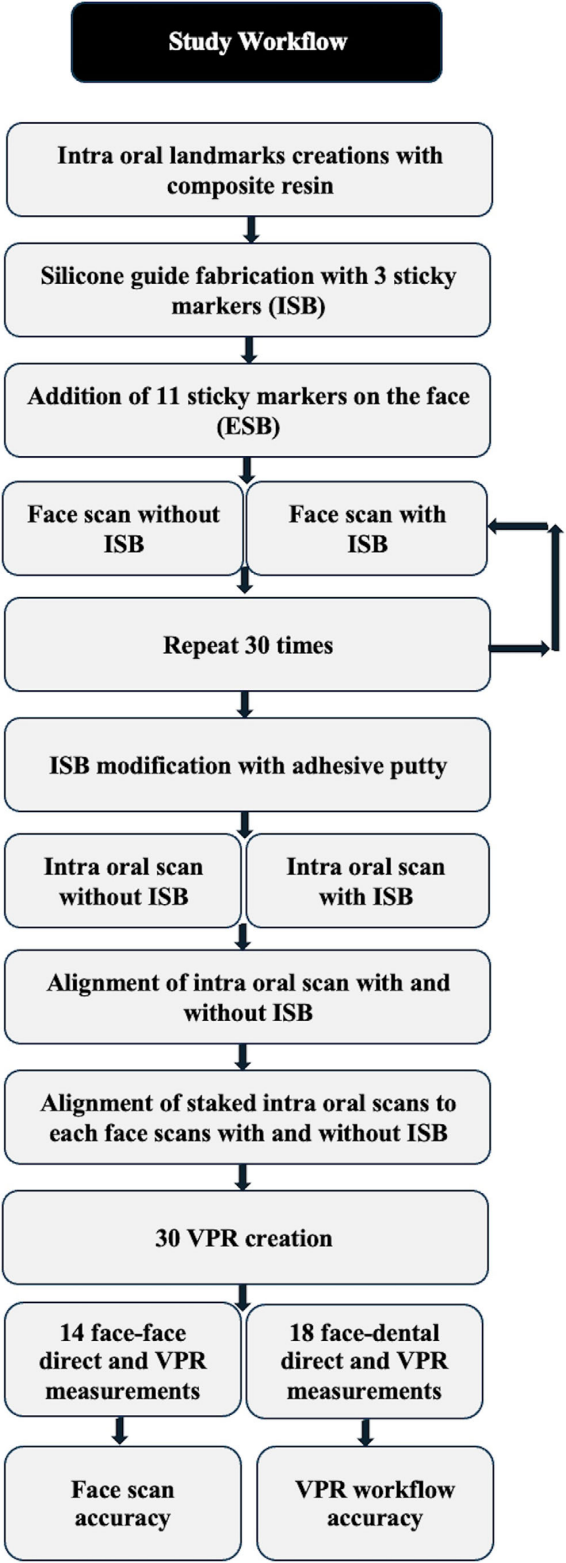
Flow chart of study workflow.

A total of 15 fiducial landmarks were selected for face‐face and face‐dental linear measurements: five sticky markers were selected as face landmarks: middle of forehead (FH), left zygion (LZ), right zygion (RZ), bridge of the nose (BN), pogonion (PO), four intraoral landmarks: the three created composite landmarks (C), (LC), (RC), and a notch on the maxillary right central incisor edge (CN), and six perioral landmarks: middle of vermilion of maxillary lip (UL), middle of vermilion of mandibular lip (LL), right commissure (RCM), left commissure (LCM), right cupid bow (RCB), and left cupid bow (LCB) (Figure 7). A total of 32 linear measurements were selected among the face, perioral, and intraoral landmarks and were performed directly on the subject (reference measurements) by using a digital caliper with 0.01 readability and 0.02 tolerance (FINO Digital Caliper; FINO GmbH, Bad Bocklet, Germany) and virtually on the VPR with the dental CAD software program (exocad; exocad GmbH). These measurements included 14 face‐face and 18 face‐dental dimension measurements (Table S1). For higher precision in identifying the landmark for measurement, the lower right angle of the cross in each sticky marker was selected for the face landmarks as the reference for the virtual and direct measurement. The other perioral and intraoral landmarks were easily identifiable. A flowchart of the study protocol is presented in Figure 8.

Accuracy is described by trueness and precision. Trueness refers to the closeness of agreement between the mean of test values and reference values; precision describes the closeness of agreement among intragroup data obtained by repetitive measurements.^18^ Trueness was evaluated by the mean absolute difference (MAD) between the virtual and direct (reference) measurements, and the standard deviation (SD) represented precision. The index for the error margin in anthropometry was evaluated with the technical error of measurement (TEM) and the error magnitude to the size of the measurement was evaluated with the relative error magnitude (REM). Measurements were repeated five times for the reference and the VPR measurements, and intra examiner reliability was evaluated with intraclass correlation coefficient. Statistical analysis was performed with statistical software package (IBM SPSS Statistics v25; IBM Corp), with α = 0.10. Data were analyzed for normality with Shapiro‐Wilk test, and one‐sample t‐test for the null hypothesis that there is no observed difference between the virtual and reference measurements (observed difference = zero) (or Wilcoxon signed rank tests if data is not normally distributed). The measurements were divided into three regions: total face, lower half face, and perioral regions (Figure 7). The accuracy of FS is represented by the face‐face measurements and the accuracy of the VPR alignment workflow is represented by the face‐dental measurements.

## RESULTS

**FIGURE 9:**
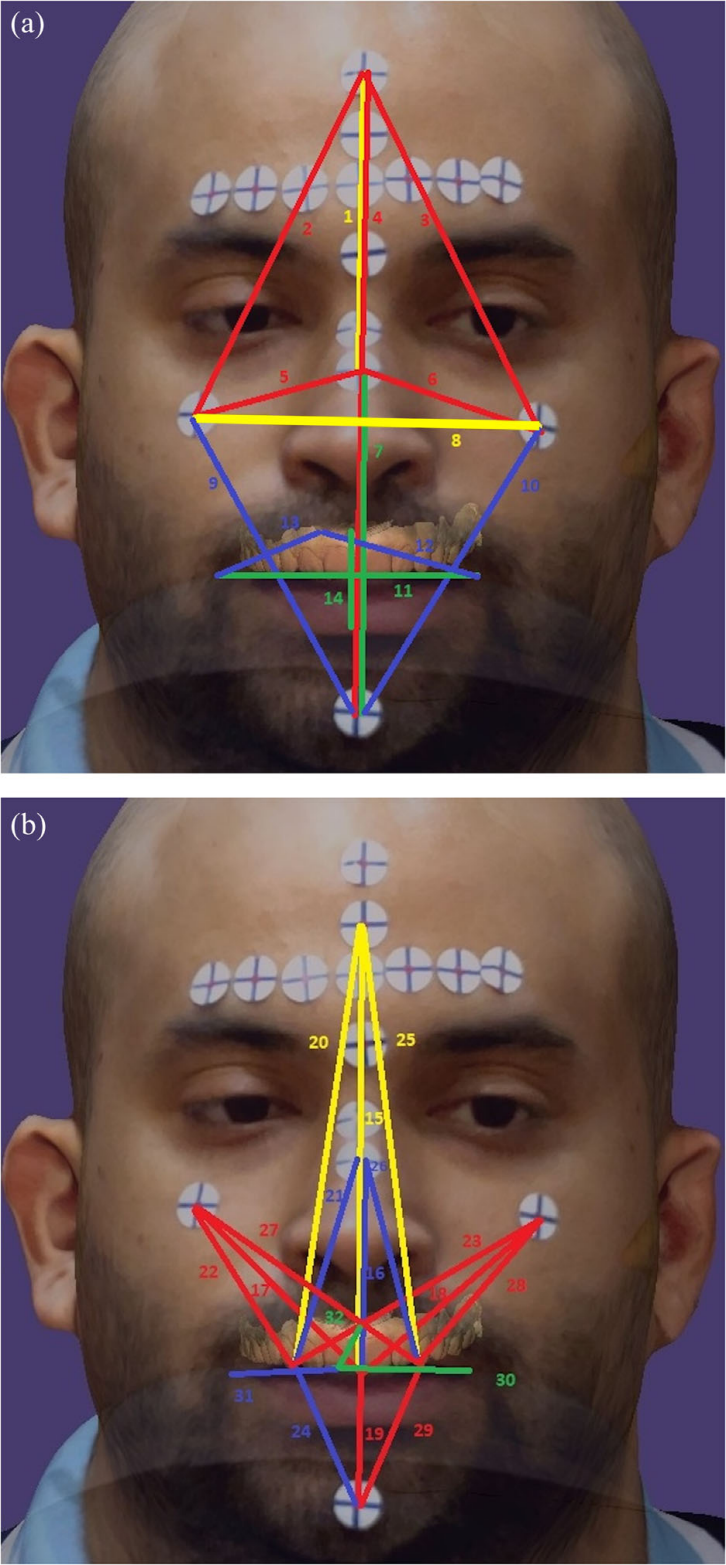
Color‐coded mean absolute difference (MAD) between VPR and reference linear measurements. (a) Face‐face measurements, (b) face‐dental measurements. Green: <1, Blue: 1–2, Red: >2–4 mm, Yellow: >4 mm.

Descriptive statistics for all measurements are presented in Table 1. Data were normally distributed except for 14 measurements. All VPR measurements were statistically significant from the reference measurements (*p* ≤ 0.006), except for BN‐PO (*p* = 0.936) and UL‐LL (*p* = 0.191) measurements, with total trueness of MAD = 2.76, and precision (SD) of 1.70. The VPR measurements were larger than the reference in only seven measurements: BN‐PO, C‐FH, C‐PO, RC‐PO, LC‐PO, CN‐LCM, and CN‐UL. FS accuracy had 2.04, 1.66, 0.80 trueness, and 1.05, 0.92, 0.91 precision for the total, lower face, and perioral regions. The VPR alignment workflow had higher MAD (lower trueness) than FS, including 3.32, 2.40, 1.21 accuracy and 2.2, 1.47, 1.2 precision for the total, lower face, and perioral regions (Table 2). Both TEM and REM were lowest for the perioral region and increased with increasing measurement distance. Also, FS accuracy had lower TEM and REM than the VPR accuracy (Table 2). Color‐coded measurements are presented in Figure 9a and b. Intra class correlation analyses showed high reliability for the actual (1.00) and virtual measurements (0.991).

**TABLE 1:** Descriptive statistics for all measurements in millimeters (mm).

Measurements		Direct (reference) Mean	VPR Mean	MAD	SD	TEM	REM	*p* ^b^
FS accuracy: Face‐face measurements								
1	FH‐BN	59.69	55.14	4.55	0.77	3.26	7.62	0.000
2	FH‐RZ	71.43	67.84	3.59	0.48	2.56	5	0.000
3	FH‐LZ	75.36	72.17	3.19	0.44	2.27	4.22	0.000
4	FH‐ PO	135.98	133.88	2.10	3.73	2.98	2.7	0.006
5	BN‐RZ	43.39	40.99	2.40	0.46	1.73	5.5	0.000
6	BN‐LZ	46.50	44.36	2.14	0.43	1.54	4.58	0.000
7	BN‐PO^a^	82.32	82.47	0.15	1.12	0.78	0.99	0.936
8	RZ‐LZ	72.25	67.65	4.60	0.49	3.27	6.3	0.000
9	RZ‐PO	83.48	81.92	1.56	1.36	1.45	2.1	0.000
10	LZ‐PO	82.98	81.95	1.03	1.76	1.42	1.9	0.002
11	RCM‐LCM	57.20	56.63	0.57	0.56	0.56	0.99	0.000
12	RCB—LCM	41.80	40.48	1.32	1.27	1.28	3.22	0.000
13	RCM‐UL	40.95	39.78	1.17	1.04	1.10	2.86	0.000
14	UL‐LL^a^	14.57	14.40	0.17	0.80	0.57	1.23	0.191
VPR workflow accuracy: Face‐dental measurements								
15	C‐FH	86.46	96.60	10.14	14.57	5.14	8.34	0.003
16	C‐BN	51.02	49.33	1.69	1.13	1.43	3.43	0.000
17	C‐RZ^a^	54.01	50.02	3.99	1.45	3.00	7.3	0.000
18	C‐LZ^a^	53.64	50.30	3.34	1.01	2.46	6.2	0.000
19	C‐PO^a^	36.31	38.58	2.27	1.78	2.03	6.5	0.000
20	RC‐FH	103.17	96.53	6.64	1.76	4.85	6.4	0.000
21	RC‐BN^a^	54.19	52.71	1.48	2.18	1.84	3.5	0.001
22	RC‐RZ^a^	44.20	40.27	3.93	1.62	3.00	9	0.000
23	RC‐LZ	64.54	60.65	3.89	1.64	2.97	6	0.000
24	RC‐PO^a^	42.07	43.96	1.89	2.01	1.93	5.1	0.000
25	LC‐FH	104.20	97.30	6.90	1.24	4.95	6.6	0.000
26	LC‐BN^a^	54.73	53.30	1.43	1.54	1.47	3	0.000
27	LC‐RZ^a^	63.09	59.32	3.77	1.28	2.81	5.8	0.000
28	LC‐LZ^a^	44.49	40.82	3.67	1.27	2.74	8.2	0.000
29	LC‐PO^a^	41.61	43.66	2.05	1.58	1.82	5.3	0.000
30	CN‐LCM	32.36	32.61	0.25	1.09	0.78	0.74	0.006
31	CN‐RCM^a^	24.82	22.98	1.84	1.10	1.51	7.41	0.000
32	CN‐UL	17.75	18.33	0.58	1.42	1.07	3.21	0.002

Abbreviations: BN, bridge of nose; C, central incisor embrasure; CN, notch on right central incisal edge; FS, face scanning; FH, middle of forehead; LC, left central and lateral incisor embrasure; LCB, left cupid bow; LCM, left commissure; LL, middle of vermilion of mandibular lip; LZ, left zygon; MAD, mean absolute difference; PO, pogonion; RC, right central and lateral incisor embrasure; RCB, right cupid bow; RCM, right commissure; REM, relative error magnitude; RZ, right zygon; SD, standard deviation; TEM, technical error of measurement; UL, middle of vermilion of maxillary lip; VPR, virtual patient representation.

^a^ Data not normally distributed (Shapiro‐Wilk test).

^b^ Statistical significance analysis with one‐sample t‐test (or Wilcoxon signed‐ranked test for not normally distributed data).

**TABLE 2:** Descriptive statistics of facial scan and VPR accuracy for total face, lower face, and perioral regions.

Direct (reference) Mean	VPR Mean	MAD	SD	TEM	REM
Facial scan accuracy: Face‐face measurements					
Region: Total face					
64.85	62.83	2.04	1.05	1.77	3.51
Region: Lower face					
56.54	55.06	1.66	0.92	1.37	2.97
Region: Perioral					
38.63	37.82	0.80	0.91	0.88	2.08
VPR workflow accuracy: Face‐dental measurements					
Region: Total face					
54.04	52.63	3.32	2.20	2.54	5.67
Region: Lower face					
45.26	43.79	2.40	1.47	2.06	5.38
Region: Perioral					
24.98	24.64	1.21	1.20	1.12	3.79

Abbreviations: MAD, mean absolute difference; REM, relative error magnitude; SD, standard deviation; TEM, technical error of measurement; VPR, virtual patient representation.

## DISCUSSION

This study investigated the accuracy of FS obtained with an accessory scanner sensor mounted on a tablet computer and also investigated the accuracy of a VPR workflow. Data showed significant differences between the reference and virtual measurements for FS and VPR measurements, therefore both null hypotheses were rejected.

Acquiring FS is a technique‐sensitive procedure, which could be further complicated by several factors, including subject movement, nose breathing, lip twitching, and eye blinking.^7^, ^13^ Room lighting conditions were optimized with a spot track device to provide optimal (6500 K) lighting conditions. Care was taken to avoid direct lighting exposure on the scanned face because this can disturb the FS process. This was dictated by the scanning software program (ItSeez3D App; ItSeez3D).

Facial scanning and IOS demand different conditions for optimal results; FS works best with flat surfaces with minimal intricate features, whereas IOS requires areas with distinctive features.^3^ Therefore, the ISB was modified after the FS by placing adhesive putty and shaped to provide intricate and distinct features.

The scanning software offers two scanning options: full body and head scans. The scanning parameters were optimized by selecting the head scan option. Also, the class 1 infrared laser beam of the accessory scanner required avoiding strong and direct lighting conditions, and shiny or black colors close to the face.^11^, ^12^ Furthermore, ring‐shaped beads were added over the center of each marker to facilitate the alignment between the intraoral scan (with transfer body) and face scan (with transfer body), since the STL file does not include color (Figure 5c).

Various types and designs of ISB and ESB were utilized in previous studies.^1^, ^2^, ^3^, ^4^ These would require manufacturer‐dependent or custom fabrication. In this study, a user‐friendly VPR protocol with a silicone guide was used. One protocol included replacing the ESB with direct placement of dot marks on the face.^5^ However, this would require a dedicated high‐end face scanner to picture the dots. In this study, a larger marker design was needed to be compatible with the accessory sensor/tablet set‐up.

The 3D scanner accessory used in this study is a range‐sensing device equipped with structured light technology, and it has been used in different health applications including foot and limb prostheses, craniofacial imaging, and surface morphology analysis.^11^, ^15^ The employed workflow for the total face region demonstrated the lowest trueness of 2.76 mm and precision of 1.70 mm in comparison to the lower face (2.05 mm trueness and 1.25 mm precision) and perioral region (0.95 mm trueness and 1.04 precision). The results in this study could be explained by the relatively short inter‐landmark distances of the perioral region compared to other distances of the face, with around 2.8% REM for the perioral measurements and 4.7% REM for the total face. Also, this was influenced by the complexity of surface features, where FS tends to be less optimized for the curved cheek surfaces. Therefore, REM tends to be higher as the inter‐landmark distances and the curvature level and intricate details increase (Figure 9). Also, the data showed that FS had lower MAD (higher trueness and precision) than VPR by almost 1 mm. The FS accuracy corresponds to the accuracy of the accessory sensor device (Structure sensor pro; Occipital Inc) and to the construction ability of the used third‐party scanning software (ItSeez3D App; ItSeez3D), whereas VPR accuracy corresponds to the combined error in FS and VPR workflow alignment process.

The actual measurement was made with a digital caliber with a validity of ± 0.01 mm and high reliability (ICC = 1.00). The validity of the actual measurements was enhanced by the placement of composite indentations for caliber stabilization, which facilitated the passive placement of the caliber without pressure, and also by using easily identifiable face landmarks. However, the VPR entailed alignment steps of the FS and IOS files by using an approximation process including manual identification of common areas. Despite having high reliability, the validity of the alignment process was jeopardized by the manual alignment of the dental CAD. In an attempt to improve the validity of this manual approximation process, the lower right angle of the blue cross was used in all except for the first alignment of files between the IOS with ISB and FS with ISB. This is because the STL file of the IOS lacks color and hence the cross would not be visible. Instead, the center of the cross, which was marked by putty, was used. Other metrology software in the industrial domains (Geomagic Control X; Artec 3D, California, USA) use automated iterative closest point algorithms for higher validity. Therefore, it seems that the VPR modules in the currently available dental CADs are primarily suited for smile design simulation rather than manufacturing purposes. Nevertheless, with advancements in digital dentistry and VPR, AI‐driven algorithms for the alignment process are expected to be employed.

There is no consensus on a classification for the reliability of FS or VPR accuracy. One study classified the reliability into four categories: highly reliable (deviation <1.0 mm), reliable (deviation 1.0 to <1.5 mm), moderately reliable (deviation 1.5 to <2 mm), and unreliable (deviation ≥2 mm).^16^ Based on this, the total accuracy in this study would be classified as highly reliable for FS and reliable for VPR for the perioral region, moderately reliable for FS and unreliable for VPR for the lower face region, and unreliable for both FS and VPR for the total face region. Therefore, the used protocol could be deemed useful for treatment planning and smile design analysis. However, further improvements are needed for more demanding prostheses manufacturing.

The results of FS accuracy in this study were higher than those reported by Knoops et al.^12^ This could be attributed to the different methodology in theat study, including investigating four scanners in comparison to a reference face scan. Yu et al.^17^ investigated the FS accuracy for three scanner devices including an accessory sensor scanner. The authors reported 0.56–1.89 mm accuracy for the accessory sensor. Also, the authors reported a tendency for increased error as the inter‐landmark distance increases, which is similar to the results in this study. Beaumont et al.^14^ investigated the FS accuracy of one industrial face scanner (M4D; Acuitaine,), and one accessory scanner (Structure sensor; Occipital Inc) and used standard anthropometric measurements of the head shape as references. The accessory Sensor proved useful for measuring both circumference and cephalic index but demonstrated a maximum error of nearly 13%. The Accessory scanner used in the present study is a later version and has higher accuracy than the one used in the study by Beaumont et al.^14^

Previous research and technique articles on VPR used different scanners, ISBs and ESBs, software, research methodologies, and VPR workflows, and reported 0.5–2.9 trueness range and 1.29–2.12 precision range.^1^, ^2^ The results of the total face region in this study are slightly higher than the reported range of trueness, but the results of the lower face and perioral regions are comparable.

Study limitations include the use of only one subject with a repetitive measure research protocol for descriptive accuracy analysis. Investigating other variables, including different face characteristics, gender, and color, is recommended in further studies. Also, one accessory scanner and workflow was used in this study. Future studies could investigate direct comparisons with other scanner devices and workflows.

## CONCLUSION

Error in face scanning increased with increased distance and intricate details as demonstrated by the linear distance measurements including the forehead and bi zygoma landmarks. VPR accuracy was lower than the FS accuracy because of the added errors in the alignment of several files during the Workflow protocol. The investigated VPR workflow might be feasible for treatment planning and smile design. However, VPR accuracy for the total and lower half of the face regions would be unreliable for the more demanding prostheses manufacturing. Adhesive markers and silicone guides could be considered viable alternatives to extraoral and intraoral transfer bodies.

## CONFLICT OF INTEREST STATEMENT

The authors declare no conflicts of interest.

## Supporting information



Supporting Information
