# Supplementary material for: Trueness and precision of facial scan and virtual patient representation workflow
Source: J Prosthodont. 2025 Jan 21;34(7):703–11. doi: 10.1111/jopr.14024 (PMC12378954; doi:10.1111/jopr.14024)
Supplement: Supplementary file 1 — Supporting Information [file JOPR-34-703-s001.docx]

Supplementary Table S1. All 32 linear measurements with 14 face-face measurements and 18 face-dental measurements.

| **FS accuracy: Face-face measurements** | | |
| --- | --- | --- |
| 1 | **FH-BN** | Middle of forehead-bridge of nose |
| 2 | **FH-RZ** | Middle of forehead-right zygon |
| 3 | **FH-LZ** | Middle of forehead-left zygon |
| 4 | **FH- PO** | Middle of forehead-pogonion |
| 5 | **BN-RZ** | Bridge of nose-right zygon |
| 6 | **BN-LZ** | Bridge of nose-left zygon |
| 7 | **BN-PO*** | Bridge of nose-pogonion |
| 8 | **RZ-LZ** | Right zygon-left zygon |
| 9 | **RZ-PO** | Right zygon-Pogonion |
| 10 | **LZ-PO** | Left zygon-Pogonion |
| 11 | **RCM-LCM** | Right commissure-left commissure |
| 12 | **RCB - LCM** | right cupid bow-left commissure |
| 13 | **RCM-UL** | Right commissure- middle of vermilion of maxillary lip |
| 14 | **UL-LL*** | Middle of vermilion of maxillary lip- middle of vermilion of mandibular lip |
| VPR workflow accuracy: Face-dental measurements | | |
| 15 | **C-FH** | Central incisor embrasure-middle of forehead |
| 16 | **C-BN*** | Central incisor embrasure-bridge of nose |
| 17 | **C-RZ*** | Central incisor embrasure-right zygon |
| 18 | **C-LZ*** | Central incisor embrasure-left zygon |
| 19 | **C-PO*** | Central incisor embrasure-pogonion |
| 20 | **RC-FH** | Right central and lateral incisor embrasure-middle of forehead |
| 21 | **RC-BN*** | Right central and lateral incisor embrasure-bridge of nose |
| 22 | **RC-RZ*** | Right central and lateral incisor embrasure-right zygon |
| 23 | **RC-LZ** | Right central and lateral incisor embrasure-left zygon |
| 24 | **RC-PO*** | Right central and lateral incisor embrasure-pogonion |
| 25 | **LC-FH** | Left central and lateral incisor embrasure-middle of forehead |
| 26 | **LC-BN*** | Left central and lateral incisor embrasure-bridge of nose |
| 27 | **LC-RZ*** | Left central and lateral incisor embrasure-right zygon |
| 28 | **LC-LZ*** | Left central and lateral incisor embrasure-left zygon |
| 29 | **LC-PO*** | Left central and lateral incisor embrasure-pogonion |
| 30 | **CN-LCM** | Notch on right central incisal edge-left commissure |
| 31 | **CN-RCM*** | Notch on right central incisal edge-right commissure |
| 32 | **CN-UL** | Notch on right central incisal edge-middle of vermilion of maxillary lip |
